# Relationship between Neuromuscular Mechanosensitivity and Chronic Neck Pain in Guitarists: A Cross-Sectional Study

**DOI:** 10.3390/ijerph18052673

**Published:** 2021-03-06

**Authors:** Valeria Estefanía Aguilar Rojas, Arisandy Flores Pluma, Daniel Pecos-Martín, Alexander Achalandabaso-Ochoa, Rubén Fernández-Matías, Patricia Martinez-Merinero, Susana Nuñez-Nagy, Tomás Gallego-Izquierdo

**Affiliations:** 1 Faculty of Medicine, Benemerita Autonoma de Puebla University, 72000 Puebla, Mexico; valeria.aguilar81293@gmail.com (V.E.A.R.); arisandy.pluma@gmail.com (A.F.P.); 2 Department of Physiotherapy and Nursing, Alcalá University, 28871 Alcalá de Henares, Spain; daniel.pecos@uah.es (D.P.-M.); susana.nunez@uah.es (S.N.-N.); tomas.gallego@uah.es (T.G.-I.); 3 Research Institute of Physical Therapy and Pain, Alcalá University, 28871 Alcalá de Henares, Spain; ruben.fernanmat@gmail.com (R.F.-M.); patricia.m.merinero@gmail.com (P.M.-M.); 4 Department of Health Sciences, Universidad de Jaén, 23071 Jaén, Spain; 5 Physiotherapy Department, Faculty of Health, Exercise and Sport, European University, 28660 Madrid, Spain

**Keywords:** pressure pain threshold, neural test, neck disability, upper extremity disability, musicians

## Abstract

Musicians frequently complain of musculoskeletal pain due to high mechanical demands, with the cervical spine being the most affected. Increased neuromuscular mechanosensitivity due to repetitive mechanical stress over time has been described in neck pain patients. Nevertheless, the association between musculoskeletal pain and neuromuscular mechanosensitivity in musicians is unknown. Therefore, the aim of this study was to analyze the relationship between neuromuscular tissue mechanosensitivity and neck pain in guitarists. Guitarists with chronic neck pain (*n* = 70) and without pain (*n* = 70) were enrolled. Pain and disability were measured by the visual analogue scale and the Neck Disability Index, respectively. The pressure pain threshold (PPT) was bilaterally measured for the upper trapezius and median nerve. Finally, the Upper limb neural test one (ULNT1) was bilaterally measured. The analyses included a 2-by-2 mixed analysis of variance, pairwise comparisons with Bonferroni correction, linear regression model, and multiple linear regression. Our data showed that chronic neck pain guitarists have a lower PPT at all locations compared to healthy guitarists. They also showed a bilateral main effect for pain for ULNT1 compared to healthy guitarists. These results were not affected by the mediator variables. Finally, a relationship between upper trapezius PPT and median nerve PPT was found.

## 1. Introduction

Musculoskeletal complaints have been identified as the most prevalent medical problem for instrumental musicians [1]. High demands on the musculoskeletal system render the musician vulnerable to developing musculoskeletal pain [2]. The lifetime prevalence can vary as much as 25.8% to 87% [3,4], and playing capacity can be reduced as much as 85% [3]. These musculoskeletal complaints in musicians are often referred to as playing-related musculoskeletal disorders (PRmDs) [3,5], which can result in serious playing-related disability and even potentially threaten performance quality as well as the musician’s quality of life [6]. Most PRmDs affect the upper body, with highest prevalence in the cervical spine and shoulder [6,7].

Neck pain is expected in up to 71% of people during their lifetime [8]. Although neck pain is primarily diagnosed as nonspecific and favorable in most people, it has been associated with decreased work productivity and daily activity limitations [9]. This leads to a high economic and social burden [10]. Different treatments have shown to be effective for non-specific neck pain, including multimodal care (exercise and manual therapy), pain education, and non-steroidal anti-inflammatory drug use [11].

Non-specific neck pain has not been associated with a specific pathological finding. Thus, other explanations about the onset of pain have been postulated. One possible explanation is the mechanosensitization of structures due to overuse. Greater mechanosensitivity of the cervical region and median nerve has been described in neck pain patients [12]. In addition, different authors have suggested that postural alterations and repetitive mechanical stress over time could play roles in the development of pain through an increase in tissue mechanosensitivity [13,14,15]. Recently, Pacheco et al. [16] showed that low-intensity mechanical stress maintained over time can potentially induce neurogenic inflammation, which could induce mechanical sensitization. Playing a musical instrument requires repetitive use of neuromuscular tissues, very often accompanied with poor posture, which increases the risk for musculoskeletal disorder [17]. In addition, musicians with cervical PRmDs have shown a higher prevalence of scapular and cervical motor control deficits [18], and they have been associated with higher muscular activity in the neck and shoulder region [19], which can lead to tissue overload.

Despite the suggested relationship between overuse of neuromusculoskeletal tissues and pain, little is known about this relationship in musicians. To our knowledge, there are no studies in the literature reporting on the link between musculoskeletal pain and mechanosensitivity alterations in musicians. Therefore, the objective of this study was to determine the possible relationship between neuromuscular tissue mechanosensitivity and neck pain in guitarists.

## 2. Materials and Methods

### 2.1. Design

This cross-sectional study was conducted according to the recommendations of Strengthening the Reporting of Observational Studies in Epidemiology (STROBE) [20]. Ethical approval was obtained from the Ethical Committee of Benemérita Universidad Autónoma de Puebla, Mexico (02/112/732). The study was conducted according to the Declaration of Helsinki.

### 2.2. Subjects

A convenience sample of guitarists was recruited through announcements at the Benemérita Universidad Autónoma de Puebla (Mexico) from June to August 2019. Before participation, all subjects signed a consent form.

The inclusion criteria were as follows: guitarists aging between 15 and 65 years and either nonspecific neck pain for at least 3 months (painful group) or absence of neck pain in the last 3 months (healthy group). The exclusion criteria were as follows: previous traumatic lesions in the neck or upper limbs, pregnant, neurological signs and/or symptoms, diagnosis of radiculopathy or cervical stenosis, bilateral symptoms in both upper limbs, previous surgery in the neck or upper limbs in the last year, systemic diseases, and having been treated with analgesics, anti-inflammatory drugs, or physical therapy in the last month.

### 2.3. Sample Size

The sample size was calculated on the basis of the main between-subject effect (presence of neck pain) of a 2-by-2 mixed analysis of variance (ANOVA). The effect size was estimated to be 0.25, with a repeated measures correlation of 0.50, 90% power, and α value of 0.05. According to the sample size calculation, 130 subjects had to be recruited. The final sample was composed of 140 subjects (70 cases and 70 controls).

### 2.4. Measurements

All measurements were carried out at the Benemérita Universidad Autónoma de Puebla by four physiotherapists with more than 10 years of experience. Evaluator 1 collected data from demographic information, including pain intensity and disability. Evaluator 2 measured pressure pain threshold (PPT), and evaluators 3 and 4 measured the range of motion during the upper limb neural test 1 (ULNT-1). Evaluators 2, 3, and 4 were blinded regarding the presence of neck pain in the subjects and their respective measurements.

#### 2.4.1. Pain and Disability

Pain intensity during the previous week was measured with a visual analogue scale (VAS), where 0 represented no pain, and 10 represented the worst pain imaginable. VAS has shown good reliability in previous studies with an intraclass correlation coefficient (ICC) ranging from 0.71 to 0.90 [21,22].

The degree of disability related to the cervical spine was measured with the *Neck Disability Index* (NDI). The NDI was transculturally adapted from English to Spanish in 2010, and it has shown good reliability (ICC = 0.98) [23]. This questionnaire ranges from 0 (no disability) to 50 (maximum degree of disability).

The degree of disability related to the upper limb was measured with the *Disabilities of the Arm, Shoulder and Hand (*DASH) questionnaire, which was transculturally adapted from English to Spanish in 2006 [24]. DASH has shown good reliability (Cronbach Alpha = 0.96). This questionnaire ranges from 0 (no disability) to 100 (maximum degree of disability).

#### 2.4.2. Pressure Pain Threshold

Mechanosensitivity was evaluated by the PPT and was measured with a hand-held algometer (Wagner Force Dial, Model FDK 20), which has a 1 cm^2^ head that records pressure in kg/cm^2^. The pressure was increased by 1 kg per second, and patients were told to indicate when the sensation changed from pressure to pain. Three measurements were taken with a 30-s rest period in between, and the mean was used for statistical analysis [25].

PPT was measured in the upper trapezius muscle, at the mid-point between the spinous process of the seventh cervical vertebrae and the lateral border of the acromion, with the subject lying in a prone position. The measurement of PPT in cervical muscles has shown good reliability, with ICC = 0.78–0.93 [25].

PPT was also measured in the median nerve at the location described by Sterling et al. [26], which has shown good reliability (ICC = 0.92–0.97). The median nerve was localized by manual palpation, and the measurement point was marked with a marker. The subject was lying in the supine position with their upper limb alongside the body and placed in external glenohumeral rotation, elbow extension, and forearm supination. The median nerve was localized at the ulnar fossa, medial and immediately adjacent to the tendon of the biceps brachii muscle [26].

#### 2.4.3. Upper Limb Neural Test One

The range of elbow extension movement during ULNT1 was measured with a digital goniometer (Digital Absolute Axis Goniometer, Baseline^®^). The axis of the goniometer was placed over the medial epicondyle of the elbow, the fixed arm was oriented towards the humeral head, and the movable arm was oriented towards the ulnar head [27]. Measurements of the elbow extension range of movement during ULNT have shown good reliability (ICC = 0.80–0.89) [28]. The subject was lying in a supine position with their head and neck in a neutral position and the contralateral upper limb alongside the body. Evaluator 3 performed the ULNT1 until the maximum tolerance was referred by the subject, or until maximum resistance was felt. Meanwhile, evaluator 4 measured the elbow extension range of motion with the digital goniometer. Two measurements were taken with a 1-min rest period in between, and the mean was used for statistical analysis.

### 2.5. Statistical Analysis

Normal distribution of the data was evaluated with the Kolmogorov–Smirnov test (*p* > 0.05). For the descriptive analysis of continuous variables, the mean and standard deviation (SD) were reported. For the categorical variables, the absolute frequencies and percentages were reported. Homogeneity of demographic variables between groups was evaluated with Student’s *t*-test for continuous variables and with Pearson’s chi-square test for categorical variables [29].

To analyze differences between groups in PPT and ULNT1 a 2-by-2 mixed ANOVA was conducted with pain (yes, no) as the between-subject factor and side (dominant, non-dominant) as the within-subject factor. Post hoc pairwise comparisons were analyzed with Student’s t-test with Bonferroni correction. Eta partial squared (η_p_^2^) was used to estimate the effect size of the main effects and interactions of the ANOVA. Cohen’s *d* was used to estimate the effect size of the pairwise comparisons [29].

To analyze (within the pain group) the relationship between the means of both sides (dominant and non-dominant) from PPT and ULNT1, stepwise linear regression models were constructed in two steps for the VAS, DASH, NDI, time with pain, time playing instrument, and time playing instrument per week measurements. In the first step, the age, height, weight, and sex were included as covariates. In the second step, all the predictor variables were included to measure the change in the coefficient of determination (R^2^) adjusted for covariates. If the change in R^2^ was statistically significant, then the standardized regression coefficients (β) were analyzed to evaluate the strength of association between each predictor and the predicted variable [29].

Stepwise multiple linear regression models were also constructed to evaluate the relationship between PPT measurements and ULNT1, and to evaluate the relationship between VAS, DASH, and NDI, with age, height, weight, and sex as covariates [29].

Cohen’s *d* effect sizes were calculated with the “effsize” package (Torchiano M, 2020) in R statistical software Version 3.5.3 (R Core Team (2019). R is a language and environment for statistical computing (R Foundation for Statistical Computing, Vienna, Austria). All other analyses were conducted using SPSS V.22 (SPSS Inc., Chicago, IL, USA). An α level of 0.05 with 95% confidence interval (CI) was assumed for all analyses.

## 3. Results

The final sample was composed of 140 subjects: 70 controls with a mean age of 25.46 (SD, 10.01) years and 70 cases with a mean age of 32.99 (SD, 10.25) years. Demographic characteristics of the subjects are presented in Table 1.

### 3.1. Pressure Pain Threshold

The 2-by-2 mixed ANOVA revealed a significant main effect for pain for the PPT measured at the upper trapezius muscle (F = 32.54, η_p_^2^ = 0.19, *p < 0.01)* and for PPT measured at the median nerve (F = 25.73, η_p_^2^ = 0.16, *p < 0.01*). There was a non-significant main effect for the side and a non-significant pain-by-side interaction (*p* > 0.05). Post hoc pairwise comparisons are presented in Table 2.

### 3.2. Upper Limb Neural Tension Test One

The 2-by-2 mixed ANOVA revealed a significant main effect for pain for the ULNT1 (F = 12.45, η_p_^2^ = 0.08, *p* < 0.01) but not for the side. There was a non-significant pain-by-side interaction. Post hoc pairwise comparisons are presented in Table 3.

### 3.3. Simple Mediation Analyses

**Figure 1:**
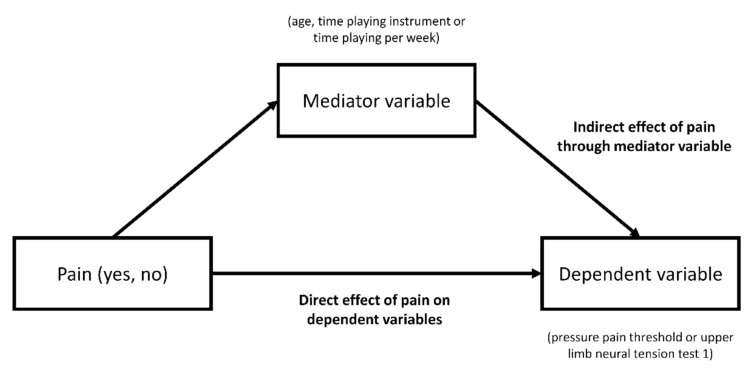
Diagram of the simple mediation model constructed.

As statistically significant between-group differences were found for age, time playing instrument, and time playing per week, simple mediation linear models (Figure 1) were constructed to evaluate if these differences affected the between-group differences for PPT and ULNT1.

Simple mediation models were constructed using the PROCESS macro version 3.4 (Andrew F. Hayes ^®^) for SPSS [30]. PPT and ULNT1 were considered the dependent variables, pain was considered the predictor variable, and age, time playing instrument, and time playing per week were considered the mediator variables. A bootstrap percentile with 5000 samples was used. The mediator variables were considered to influence the relationship between pain and the dependent variables if the indirect effect of pain through mediator variables was statistically significant.

There were no statistically significant indirect effects of pain through mediator variables (Table 4).

There were no statistically significant indirect effects of pain through mediator variables (Table 4).

### 3.4. Multiple Regression Analyses for Tissue Mechanosensitivity

Multiple regression analyses did not find an association between the predictor variables and PPT measured at the upper trapezius muscle and at the median nerve, and the ULNT1 (Table 5).

### 3.5. Relationship between Pain and Disability

Multiple regression analyses revealed that NDI and DASH significantly predicted VAS (R^2^ change = 0.14, *p* < 0.01). Analyses of the standardized regression coefficients revealed that only NDI was significantly associated with VAS (β = 0.38, *p* < 0.01). There was a non-significant relationship between NDI and DASH (R^2^ change = 0.01, *p* = 0.87) (Table 6).

### 3.6. Relationship between Pressure Pain Threshold and Upper Limb Neural Tension Test One

Multiple regression analyses revealed that PPT measured at the upper trapezius muscle and median nerve did not predict ULNT1 (R^2^ change = 0.03, *p* = 0.12). However, there was an association between PPT measured at the upper trapezius muscle and median nerve (R^2^ change = 0.46, *p* < 0.01; β = 0.70, *p* < 0.01) (Table 6).

## 4. Discussion

The aim of this investigation was to study the link between musculoskeletal pain and mechanosensitivity alterations in guitarists with chronic neck pain. The main result of this study is the presence of a relationship between musculoskeletal pain and mechanosensitivity, where guitarists with chronic neck pain have increased mechanosensitivity of muscles and neural structures. 

### 4.1. Pressure Pain Threshold

It has been shown that playing an instrument can lead to PRmDs (41–93% for professionals and 67.8% for amateurs) due to repetitive actions or sustained positions [31], which could lead to an increase in mechanosensitivity of several tissues. Recent studies have shown a positive association between pain and mechanosensitivity [12,32,33]. Our results are in agreement with those of López-de-Uralde-Villanueva et al. [12], as we found that, compared to healthy controls, patients with chronic neck pain showed a significant increase in mechanosensitivity measured as a decrease in PPT at the upper trapezius muscle and at the median nerve. This increase in mechanosensitivity could be justified by the overload proposed as a consequence of sustained positions of the upper limb when playing the guitar while moving the forearm, wrist, and/or fingers repeatedly. This situation keeps the upper trapezius contracted and could constrain blood flow, facilitating a chemical sensitization process [34], which could secondarily decrease the PPTs. Furthermore, repetitive movements of forearm, wrist, and/or fingers while playing could lead to neurogenic inflammation, as observed in an animal model [35], which could facilitate nerve sensitization and lead to a decrease in PPTs observed at the median nerve. Nevertheless, pain seems to play a prominent role in the degree of mechanosensitization since significant differences have been observed between mechanosensitivity in asymptomatic subjects who perform repetitive activities and symptomatic subjects who perform the same activities [16].

### 4.2. Upper Limb Neural Tension Test One

An increase in neural mechanosensitivity has been related to a decrease in tolerance to compression and also to strained positions [36]. Our findings support this conclusion, as we found that patients with chronic neck pain reported a significant increase in adverse neural tension from the median nerve when compared to healthy controls. In addition, in guitarists with chronic neck pain, differences between dominant and non-dominant sides were not reported. This could suggest that sensitivity is not just a peripheral process but a central process, which could lead to avoidance behavior and, in turn, could increase the mechanical load contributing to maintaining sensitivity [37]. This situation could contribute to an elevated and anteriorized shoulder position, which in turn will contribute to overdemand in the upper trapezius muscle. 

### 4.3. Simple Mediation Analyses

The differences in mechanosensitivity in our population could be explained by the between-group differences (age, years practicing, and number of hours practicing a week), since patients with chronic neck pain were significantly older, had been playing for more years, and spent more hours playing per week. Nevertheless, our mediation linear model showed these differences did not influence the relationship between pain and mechanosensitivity measured via algometry at the upper trapezius and the median nerve, or goniometry of the ULNT1.

### 4.4. Multiple Regression Analyses for Tissue Mechanosensitivity

Multiple regression analyses showed there was no association between pain, disability or time in pain, and PPTs measured at the upper trapezius muscle and at the median nerve, and the ULNT1. These findings could be due to the dimensional differences between the variables; while mechanosensitivity is a unidimensional variable, pain and disability are multidimensional variables. 

### 4.5. Relationship between Pain and Disability

We found a positive relationship between neck disability and neck pain, but not between upper extremity disability and neck pain. In addition, we found no association between neck disability and upper extremity disability. This indicates guitarists with neck pain should be assessed for neck disability since this will establish the magnitude of neck pain and its relation with functionality. However, our data suggest that assessing upper limb disability does not provide additional information on neck pain or disability. This finding is contrary to McLean et al. [38], as they found an association between neck and arm disability in a population of those reporting neck pain. We consider this finding vital in the clinical setting because it guides clinicians in evaluating a population with chronic neck pain.

### 4.6. Relationship between Pressure Pain Threshold and Upper Limb Neural Tension Test One

In our group of guitarists with neck pain, we found a significant, positive relation between PPTs in the upper trapezius and median nerve, while no association was found between PPTs in the upper trapezius and median nerve, and the ULNT1. These results, which a priori seem contradictory, could be explained by the differences in the nature of mechanical stressors; while PPTs are determined through pression, ULNT1 is determined through stretching of the nerve. Furthermore, Nee et al. [39] confirmed the importance of the joint movement order when performing neurodynamic tests, as the first joint involved would be subjected to increased nerve strain for a longer time. In ULNT1, the last parameter is elbow extension. Thus, it is possible that when ULNT1 finished, the time the median nerve was under strain may not be long enough to sensitize it at the ulnar fossa, the place where PPT of the median nerve was performed.

### 4.7. Limitations

This study has several limitations. The cross-sectional design only allows us to establish associations that require further testing. The studied population was specific, so extrapolation of the outcomes to the general musician population should be done with caution. Furthermore, we employed a non-probability sampling method, which also means extrapolation of the results should be done with caution. Finally, when comparing the PPT of the median nerve with the ULNT1, variations be considered where the elbow extension is performed at the beginning of the test. For future research, when comparing median nerve PPT and ULTN1, modification of ULNT1 should be considered in order to guarantee that strain at the ulnar fossa is applied for the longest time. 

## 5. Conclusions

On the basis of the results obtained from the present study, it would be possible to conclude a relationship between musculoskeletal pain and mechanosensitivity in guitarists with chronic neck pain. This relation was present for muscle and neural tissues, both on the dominant and non-dominant sides. Furthermore, not only were decreased PPTs at the upper trapezius muscle and median nerve observed, but adverse neural tension was also observed from the median nerve. Finally, we found an association between neck pain and neck disability, but no association was found between neck pain and upper extremity disability or between neck disability and upper extremity disability. 

## Figures and Tables

**Table 1:** Characteristics of the subjects (*n* = 140).

Characteristic *	Healthy (*n* = 70)	Neck Pain (*n* = 70)	*p*-Value
Age, years	25.46 (10.01)	32.99 (10.25)	< 0.01
Weight, kg	69.10 (11.92)	69.60 (8.85)	0.78
Height, cm	164.24 (7.13)	165.63 (5.23)	0.19
BMI, kg/m^2^	25.63 (4.11)	25.38 (3.09)	0.66
Time sleeping, hours	6.60 (1.27)	6.27 (1.08)	0.10
Time playing instrument, years	7.11 (7.15)	15.26 (9.28)	< 0.01
Time playing per week, hours	10.63 (6.42)	20.18 (7.80)	< 0.01
Time with pain, months	-	19.23 (16.60)	
VAS pain, cm	-	3.94 (1.61)	
DASH	-	52.01 (10.57)	
DASH, sport activities	-	5.69 (2.18)	
NDI	-	27.31 (8.75)	
Sex women, *n* (%)	12 (17.1)	12 (17.1)	1.00
Dominant side R, *n* (%)	68 (97.1)	64 (91.4)	0.28
Physical exercise ^#^, *n* (%)	14 (20)	10 (14.3)	0.37

* Data are presented as mean (standard deviation) unless otherwise specified. ^#^ Subjects were considered to practice physical exercise if they did it regularly (weekly), and this was measured as a dichotomous variable (yes, no). Abbreviations: BMI, body mass index; VAS, visual analogue scale; DASH, Disabilities of the Arm, Shoulder and Hand; NDI, Neck Disability Index; R, right.

**Table 2:** Between-group differences in pressure pain threshold measurements (kg/cm^2^).

Location of Measurement *	Healthy (*n* = 70)	Neck Pain (*n* = 70)	Between-Group Differences, Mean (95% CI)	Effect Size (95% CI)
Upper trapezius, dominant side	4.65 (1.82)	3.19 (1.28)	−1.46 ^‡^ (−1.99, −0.93)	0.93 (0.57, 1.28)
Upper trapezius, non-dominant side	4.35 (1.71)	3.12 (1.06)	−1.23 ^‡^ (−1.71, −0.76)	0.87 (0.52, 1.22)
Median nerve, dominant side	4.00 (1.52)	2.79 (1.44)	−1.21 ^‡^ (−1.70, −0.71)	0.82 (0.47, 1.16)
Median nerve, non-dominant side	4.08 (1.73)	2.83 (1.52)	−1.25 ^‡^ (−1.79, −0.70)	0.77 (0.42, 1.11)

* Data are presented as mean (standard deviation) unless otherwise specified. ^‡^ Statistically significant (*p* < 0.01). Abbreviations: CI, confidence interval.

**Table 3:** Between-group differences in the upper limb neural tension test measurements (degrees).

ULNT1 *	Healthy (*n* = 70)	Neck Pain (*n* = 70)	Between-Group Differences, Mean (95% CI)	Effect Size (95% CI)
Dominant side	−18.21 (6.75)	−22.84 (11.14)	4.63 ^‡^ (1.55, 7.71)	0.50 (0.16, 0.84)
Non-dominant side	−17.79 (4.45)	−22.55 (10.77)	4.76 ^‡^ (2.01, 7.52)	0.58 (0.24, 0.92)

* Data are presented as mean (standard deviation) unless otherwise specified. ‡ Statistically significant (*p* < 0.01). Abbreviations: ULNT1, upper limb neural test 1; CI, confidence interval.

**Table 4:** Indirect effects of pain on the pressure pain threshold and neural tension test through mediator variables.

Variable *	Age	Time Playing Instrument	Time Playing Per Week
PPT UT dominant side	0.06 (−0.13, 0.47)	0.10 (−0.33, 0.45)	−0.05 (−0.34, 0.32)
PPT UT non-dominant side	−0.03 (−0.23, 0.24)	0.17 (−0.17, 0.54)	0.03 (−0.23, 0.41)
PPT MN dominant side	−0.05 (-0.30, 0.23)	0.11 (−0.24, 0.44)	−0.07 (−0.38, 0.34)
PPT MN non-dominant side	−0.06 (−0.20, 0.50)	−0.03 (−0.53, 0.40)	−0.05 (−0.40, 0.43)
ULNT1 dominant side	0.01 (−0.17, 0.18)	−0.09 (−0.33, 0.12)	−0.15 (−0.41, 0.03)
ULNT1 non-dominant side	0.37 (−0.89, 2.05)	−1.17 (−3.19, 0.38)	−0.76 (−2.69, 0.71)

* Values are effect sizes (95% confidence interval). Abbreviations: PPT, pressure pain threshold; UT, upper trapezius; MN, median nerve; ULNT1, upper limb neural test 1.

**Table 5:** Multiple linear regression analyses for prediction of pressure pain threshold and neural tension test.

	Model R^2^	Step 2 R^2^ Change	*p*-Value
**Pressure pain threshold, median nerve (predicted)**			
Step 1	0.15		
Step 2	0.22	0.07	0.50
**Pressure pain threshold, upper trapezius (predicted)**			
Step 1	0.17		
Step 2	0.28	0.11	0.21
**Upper limb neural test 1 (predicted)**			
Step 1	0.09		
Step 2	0.16	0.07	0.56

Step 1: age, height, weight, and sex entered in the model. Step 2: visual analogue scale, Disabilities of the Arm, Shoulder and Hand questionnaire, Neck Disability Index, time with pain, time playing instrument, and time playing instrument per week entered in the model.

**Table 6:** Multiple linear regression analyses for the relationship between VAS, NDI, DASH, PPT, and ULNT1.

	Model R^2^	Step 2 R^2^ Change	*p*-Value
**VAS predicted by NDI and DASH**			
Step 1	0.08		
Step 2	0.22	0.14	< 0.01
**NDI predicted by DASH**			
Step 1	0.13		
Step 2	0.14	0.01	0.87
**ULNT1 predicted by PPT-UT and PPT-MN**			
Step 1	0.07		
Step 2	0.10	0.03	0.12
**PPT-UT predicted by PPT-MN**			
Step 1	0.09		
Step 2	0.55	0.46	< 0.01

Step 1: age, height, weight, and sex entered in the model. Step 2: predictor variables specified within the table for each analysis entered in the model. Abbreviations: VAS, visual analogue scale; NDI, neck disability index; DASH, Disabilities of the Shoulder, Arm and Hand; ULNT1, upper limb neural test 1; PPT-UT, pressure pain threshold of upper trapezius muscle; PPT-MN, pressure pain threshold of median nerve.

## Data Availability

The data presented in this study are available on request from the corresponding author. The data are not publicly available due to privacy reasons.
